# Anti-U3 ribonucleoprotein antibody-positive inflammatory myopathy: a case report

**DOI:** 10.1186/s13256-016-0941-4

**Published:** 2016-06-09

**Authors:** Ken-ya Murata, Kumiko Nakatani, Mika Yananeki, Ichiro Nakanishi, Hidefumi Ito

**Affiliations:** Department of Neurology, Wakayama Medical University, 840-1 Kimii-dera, Wakayama, Wakayama 641-8510 Japan

**Keywords:** Anti-U3 RNP antibody, Polymyositis, Cricopharyngeal bar, Steroid therapy

## Abstract

**Background:**

The discovery of myositis-specific autoantibodies and myositis-associated autoantibodies has led to a new serological classification. Human U3 RNP, which consists of the U3 small nucleolar RNA and anti-U3 RNP antibody, is directed against one of the subunits. Anti-U3 RNP antibodies have been detected in 5–8 % of patients with systemic sclerosis, and antibody-positive patients with systemic sclerosis have shown more frequent skeletal muscle involvement than that of antibody-negative patients with systemic sclerosis.

**Case presentation:**

A 74-year-old Japanese man positive for anti-U3 RNP antibody was referred to our hospital because of gait disturbance and dysphagia. His serum myoglobin and creatine kinase levels were elevated, and myopathic changes were observed in his proximal legs by needle electromyography. A muscle biopsy was performed at the quadriceps femoris muscle, which showed high signal intensity on fat-suppressed and T2-weighted magnetic resonance images. The patient was diagnosed with probable polymyositis because CD8-positive lymphocytes had invaded only the endomysium and not into the muscle fibers. Severe proliferation of the interstitial connective tissue and edematous changes were observed. Oral prednisolone therapy was started, and the patient’s muscle weakness of the proximal limbs improved remarkably within 1 month. Dysphagia caused by incomplete function of the cricopharyngeal muscle persisted for 5 years.

**Conclusions:**

Our findings indicate that mild muscle weakness with steroid-resistant dysphagia may be a clinical feature of patients with anti-U3 RNP antibody-positive inflammatory myopathy.

## Background

The inflammatory myopathies are classified into three major subsets (dermatomyositis, polymyositis, and inclusion body myositis) based on clinical, histopathological, immunological, and demographic criteria. The discovery of myositis-specific and myositis-associated autoantibodies has led to a new serological classification. Human U3 RNP consists of the U3 small nucleolar RNA and at least six protein subunits. One of the subunits, fibrillarin, is a 34 kDa basic protein and is considered to be the main antigenic determinant. The presence of anti-U3 RNP (antifibrillarin) antibodies is highly specific to systemic sclerosis (SSc) and is associated with skeletal muscle disease [1,2]. Here, we report a case of a patient with anti-U3 RNP antibody positivity who showed the symptoms of inflammatory myopathy, but not those of SSc.

## Case presentation

A 74-year-old Japanese man was referred to our hospital for gait disturbance and dysphagia. He had been diagnosed with prostate cancer (T2bN0M0) at 70 years old and had been treated by linear accelerator (70 Gy), followed by endocrine therapy. He had had a slightly elevated creatine kinase (CK) level (464 IU/L) in a medical examination when he was 72 years old, but he remained asymptomatic. The patient had noticed difficulty in standing up from a chair and swallowing solid foods 18 months before referral to our hospital. He had a high tendency to fall and had noticed difficulty in climbing the stairs starting from 6 months before referral to our hospital.

### Condition at initial presentation

The patient’s blood pressure was 132/66 mmHg, his pulse rate was 66 beats/minute and regular, his body temperature was 36.7 °C, and his weight was 49 kg (with a 6-kg weight loss in the past year). His heart and breath sounds were normal. No skin sclerosis or Raynaud’s phenomenon was observed. His higher cerebral function revealed that he was alert and well-oriented. His mental status was normal, and his cranial nervous system appeared to be intact. Muscle atrophy was noted in the proximal parts of his upper and lower extremities, and manual muscle testing showed decreases to level 4 in his proximal upper limbs and level 3 in his proximal lower limbs. His muscle tone and deep tendon reflexes were within normal ranges. No abnormal findings were observed in his sensory, cerebellar, and autonomic nervous systems.

### Laboratory findings

The patient’s hematology examination revealed no abnormal findings. His serum CK, aspartate aminotransferase, alanine aminotransferase, aldolase, and myoglobin levels were elevated. His KL-6 level was within normal limits (212 U/L). All of his tumor markers were negative. As for his autoimmune systems, his serum antinuclear antibody (5120-fold) titer was elevated, but his other autoantibodies were negative. Only his anti-U3 RNP antibodies were positive; his myositis-specific autoantibodies and myositis-associated autoantibodies were negative (Table 1). For assessment, a commercially available line blot test kit (Myositis and Systemic Sclerosis Profile Euroline Blot test kit; Euroimmun, Lübeck, Germany) was used according to the manufacturer’s protocols.

**Table 1:** Laboratory data

Test	Value
Hematology	
White blood cells	5700/μl
Red blood cells	441 × 10^4^/μl
Hemoglobin	13.7 g/dl
Platelet count	18.9 × 10^4^/μl
Chemistry	
Total protein	7.7 g/dl
CK	1045 IU/L
AST	54 IU/L
ALT	75 IU/L
Aldolase	21.6 IU/L
Myoglobin	386 mg/dl
Autoantibody	
Antinuclear	5120-fold
Anti-SSA/Ro	(−)
Anti-SSB/La	(−)
Anti-MPO-ANCA	(−)
Anti-RNP3-ANCA	(−)
Anti-Smith	(−)
Anti-ssDNA	(−)
Anti-dsDNA	(−)
Anti-Scl-70	(−)
Anti-centromere	(−)
Anti-RF	(−)
Anti-CCP	(−)
Myositis-specific autoantibody	
Anti-Jo-1	(−)
Anti-PL-7	(−)
Anti-PL-12	(−)
Anti-EJ	(−)
Anti-OJ	(−)
Anti-SRP	(−)
Myositis-associated autoantibody	
Anti-U1 RNP	(−)
Anti-Ku	(−)
Anti-PM-Scl	(−)
Anti-U3 RNP	(+)

*CK* creatine kinase, *AST* aspartate aminotransferase, *ALT* alanine aminotransferase, *MPO-ANCA* myeloperoxidase antineutrophil cytoplasmic antibody, *PR3-ANCA* proteinase 3 antineutrophil cytoplasmic antibody, *SRP* signal recognition particle, *dsDNA* double-stranded DNA, *ssDNA* single-stranded DNA, *RF* rheumatoid factor, *CCP* cyclic citrullinated peptide, *Scl-70* DNA topoisomerase I, *Jo-1* hystidyl-tRNA synthetase, *PL7* threonyl-tRNA synthetase, *PL12* alanyl-tRNA synthetase, *EJ* glycyl-tRNA synthetase, *OJ* isoleucyl-tRNA synthetase, *U1 RNP* U1-ribonucleoprotein, *U3 RNP* anti-U3-ribonucleoprotein

**Fig 1:**
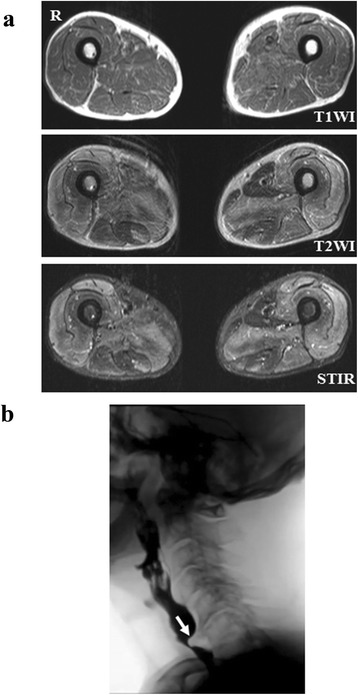
**a** Magnetic resonance imaging findings in the femoral muscles. T2-weighted and short tau inversion recovery magnetic resonance imaging scans show high signal intensity in both the flexors and extensors of the thigh muscles. **b** Videofluoroscopic study using paste barium. Pharyngeal muscle propulsion (cricopharyngeal bar) was observed (*arrow*)

**Fig. 2:**
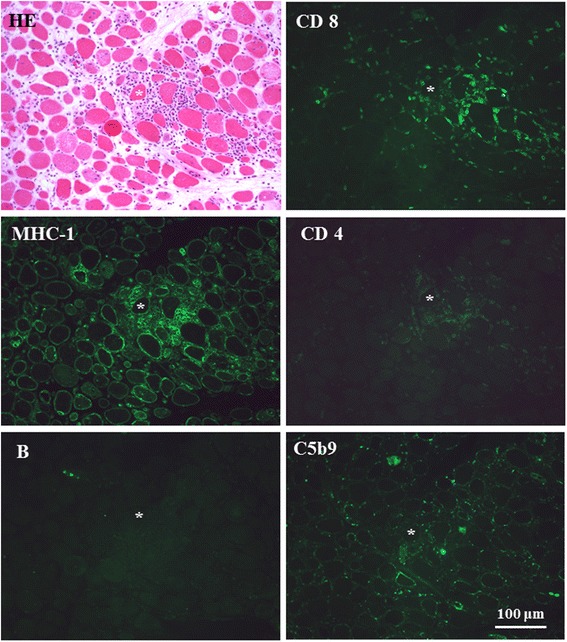
Transverse serial frozen sections of a muscle biopsy specimen from the right side quadriceps femoris muscles, stained with hematoxylin and eosin (HE) and monoclonal antibodies against CD8-positive T cells (CD8), human histocompatibility complex type 1 (MHC-1), CD4-positive T cells (CD4), B cells, and complement components (C5b9). The MHC-1-positive non-necrotic muscle fiber (*) was surrounded with CD8-positive T lymphocytes that invaded along the perimysium. No inflammatory cell infiltration into the muscle fibers can be seen

The patient’s electrocardiogram showed no remarkable findings. Upper gastrointestinal tract endoscopy showed no abnormalities such as reflux esophagitis. A computed tomographic scan showed no interstitial pneumonic or malignant findings. Needle electromyography of the patient’s proximal legs demonstrated myopathic changes without denervation potentials. T2-weighted and short tau inversion recovery magnetic resonance imaging scans revealed high signal intensity in both the flexors and extensors of the thigh muscles (Fig. 1a). Videofluoroscopic examination of the patient’s swallowing showed poor tongue movements in the oral stage and impaired opening of the esophageal muscle, as well as a cricopharyngeal bar on the posterior pharyngeal wall in the pharyngeal stage (Fig. 1b). In the esophageal stage, no obstruction, retention of the contrast material in the lower esophagus, or impaired opening of the lower esophageal sphincter were observed. Muscle biopsy specimens from the patient’s quadriceps femoris muscle showed round muscle fibers of various sizes, marked proliferation of connective tissue, and edematous changes. No perifascicular atrophy was observed. The main inflammatory cells invading the endomysium were CD8-positive T lymphocytes surrounding major histocompatibility complex (MHC) class I-positive, non-necrotic muscle fibers. No lymphocyte invasion into the muscle fibers or deposition of complement along the vascular wall was observed (Fig. 2).

### Course

Oral prednisolone (1 mg/kg/day) therapy was started, and the patient’s muscle strength of upper and lower extremities normalized within 1 month, along with his serum CK, aldolase, and myoglobin levels. Although a videofluoroscopic swallowing examination showed improvement of tongue movements during the oral stage, impaired function of the cricopharyngeal muscle and presence of the cricopharyngeal bar persisted. The patient has been followed on a regimen of prednisolone 5 mg/day for 5 years, and no relapse of the muscle weakness has been observed, except for dysphagia.

## Discussion

We present a case of a 74-year-old man who had mild proximal muscle weakness, as well as dysphagia due to incomplete opening of the cricopharyngeal muscle. In this patient, laboratory data showed elevated serum levels of CK, myoglobin, and aldolase while myopathic changes in the proximal legs were observed by needle electromyography. Although CD8-positive T lymphocytes were surrounding the MHC-1-positive, non-necrotic muscle fibers, no lymphocyte invasion into the muscle fibers was observed. On the basis of these clinical features, we diagnosed our patient as having probable polymyositis [3,4].

This patient showed the presence of anti-U3 RNP antibodies but did not fulfill the other diagnostic criteria for SSc [5]. Koenig *et al.* reported heterogeneity of autoantibodies in 100 patients with autoimmune myositis. Fourteen patients (14 %) were positive for anti-U3 RNP antibodies. All except one of these patients (93 %) had other autoantibodies, including anti-Ku, anti-Ro, and anti-Jo-1 antibodies [6]. Raynaud’s phenomenon and lung involvement are associated with anti-U3 RNP antibody-positive myositis. Thus, patients with myositis who are anti-U3 RNP-positive usually show overlap syndrome. Therefore, patients with only myositis who are positive for anti-U3 RNP antibody alone are extremely rare.

Oh *et al.* conducted a retrospective review of medical records of 783 patients with inflammatory myopathy and found that 13 patients had dysphagia as the initial presenting symptom (11 patients with inclusion body myositis, 1 patient with polymyositis, and 1 patient with overlap syndrome) [7]. Dysphagia is serious and at times problematic for patients with inflammatory myopathy. Videofluoroscopic swallowing examination of our patient revealed a cricopharyngeal bar, which represents impaired opening of the cricopharyngeal muscle [8]. Dysphagia was an initial symptom in our patient. After the initiation of oral steroid therapy (40 mg/day), his serum CK normalized rapidly and his muscle strength recovered within 1 month. Currently, the patient is being followed on a regimen of prednisolone 5 mg/day, and no relapse of his muscle weakness has been observed, except for dysphagia. Steroid therapy was effective in improving his muscle weakness, but not his dysphagia. His cricopharyngeal muscle damage was more severe than his skeletal muscle disturbance. Measurement of anti-U3 RNP antibodies has been performed by RNA immunoprecipitation, which is not a popular technique. Using other methods such as line immune assay to examine the positivity of the anti-U3 RNP antibody in inflammatory myopathy is more feasible.

## Conclusions

Our findings indicate that mild muscle weakness with steroid-resistant dysphagia may be a clinical feature of patients with only anti-U3 RNP antibody-positive inflammatory myopathy. Further observational studies are needed to confirm these findings.

## Abbreviations

RNP, ribonucleoprotein; Scl-70, DNA topoisomerase I; Jo-1, hystidyl-tRNA synthetase; PL7, threonyl-tRNA synthetase; PL12, alanyl-tRNA synthetase; EJ, glycyl-tRNA synthetase; OJ, isoleucyl-tRNA synthetase; U1 RNP, U1-ribonucleoprotein; U3 RNP, anti-U3-ribonucleoprotein

## References

[1] Baserga SJ, Yang XD, Steitz JA. An intact Box C sequence in the U3 snRNA is required for binding of fibrillarin, the protein common to the major family of nucleolar snRNPs. EMBO J. 1991;10:2645–51. 10.1002/j.1460-2075.1991.tb07807.x PMC452965 1714385

[2] Okano Y, Steen VD, Medsger Jr TA. Autoantibody to U3 nucleolar ribonucleoprotein (fibrillarin) in patients with systemic sclerosis. Arthritis Rheum. 1992;35:95–100. 10.1002/art.1780350114 1731817

[3] Dalakas MC, Hohlfeld R. Polymyositis and dermatomyositis. Lancet. 2003;362:971–82. 10.1016/S0140-6736(03)14368-1 14511932

[4] Hoogendijk JE, Amato AA, Lecky BR, Choy EH, Lundberg IE, Rose MR, et al. 119th ENMC international workshop: trial design in adult idiopathic inflammatory myopathies, with the exception of inclusion body myositis, 10–12 October 2003, Naarden, The Netherlands. Neuromusc Disord. 2004;14:337–45. 10.1016/j.nmd.2004.02.006 15099594

[5] van den Hoogen F, Khanna D, Fransen J, Johnson SR, Baron M, Tyndall A, et al. Classification criteria for systemic sclerosis: an American College of Rheumatology/European League against Rheumatism collaborative initiative. Arthritis Rheum. 2013;2013(65):2737–47. 10.1002/art.38098 PMC3930146 24122180

[6] Koenig M, Fritzler MJ, Targoff IN, Troyanov Y, Senecal JL. Heterogeneity of autoantibodies in 100 patients with autoimmune myositis: insights into clinical features and outcomes. Arthritis Res Ther. 2007;9:R78. 10.1186/ar2276 PMC2206383 17688695

[7] Oh TH, Brumfield KA, Hoskin TL, Stolp KA, Murray JA, Bassford JR. Dysphagia in inflammatory myopathy: clinical characteristics, treatment strategies, and outcome in 62 patients. Mayo Clin Proc. 2007;82:441–7. 10.4065/82.4.441 17418072

[8] Murata KY, Kouda K, Tajima F, Kondo T. A dysphagia study in patients with sporadic inclusion body myositis (s-IBM). Neurol Sci. 2012;33:765–70. 10.1007/s10072-011-0814-y 21993833

